# Early Mortality, Cardiovascular, and Renal Diseases in Women's Lives Following Hypertensive Disorders of Pregnancy: The Prospective Nationwide Study CONCEPTION

**DOI:** 10.1161/JAHA.123.033252

**Published:** 2024-04-02

**Authors:** Grégory Lailler, Clémence Grave, Amélie Gabet, Pierre Joly, Nolwenn Regnault, Catherine Deneux‐Tharaux, Vassilis Tstsaris, Geneviève Plu‐Bureau, Sandrine Kretz, Jacques Blacher, Valérie Olie

**Affiliations:** ^1^ Santé Publique France Saint‐Maurice France; ^2^ Université Paris Est Créteil France; ^3^ Centre Inserm U1219—Bordeaux Population Health Université de Bordeaux—ISPED Bordeaux France; ^4^ Obstetrical Perinatal and Pediatric Epidemiology Research Team EPOPé, Centre for Epidemiology and Statistics Sorbonne Paris Cité (CRESS), INSERM Paris France; ^5^ Université Paris Cité Paris France; ^6^ Maternité Port‐Royal FHU PREMA, Assistance Publique Hôpitaux de Paris, Hôpital Cochin Paris France; ^7^ Unité de Gynécologie Médicale APHP, Hôpital Port‐Royal Cochin Paris France; ^8^ Centre de Diagnostic et de Thérapeutique Hôtel Dieu, AP‐HP Paris France

**Keywords:** cardiovascular diseases, cardiovascular risk factors, epidemiology, gestational hypertension, hypertensive disorders of pregnancy, preeclampsia, Preeclampsia, Cardiovascular Disease, Epidemiology, Vascular Disease, Coronary Artery Disease

## Abstract

**Background:**

We aimed to evaluate the impact of hypertensive disorders of pregnancy occurrence, recurrence, onset time, and severity on mortality and on a wide range of cardiovascular outcomes in France.

**Methods and Results:**

CONCEPTION (Cohort of Cardiovascular Diseases in Pregnancy) is a French nationwide prospective cohort using data from the National Health Data System. We included all women in CONCEPTION with no history of a cardiovascular event who delivered in France for the first time between 2010 and 2018 (N=2 819 655). Hypertensive disorders of pregnancy and cardiovascular outcomes during the study follow‐up were identified using algorithms combining *International Classification of Diseases, Tenth Revision* (*ICD‐10*) coded diagnoses during hospitalization and purchases of medication between 2010 and 2021. We fitted Cox models with time‐varying exposure to assess the associations of hypertensive disorders of pregnancy with mortality and cardiovascular events. Women with gestational hypertension had a 1.25‐ to 2‐fold higher risk of stroke, acute coronary syndrome, peripheral arterial disease, pulmonary embolism, and chronic kidney disease, and a 2‐ to 4‐fold higher risk of rhythm and conduction disorder and heart failure. Women with preeclampsia had a 1.35‐ to 2‐fold higher risk of rhythm or conduction disorder and pulmonary embolism during follow‐up; a 2‐ to 4‐fold higher risk of stroke, acute coronary syndrome, and peripheral arterial disease; and a 7‐ to 9‐fold higher risk of heart failure and chronic kidney disease. They were 1.8 times more likely to die and 4.4 times more likely to die of cardiovascular causes.

**Conclusions:**

Hypertensive disorders of pregnancy drastically increase the risk of mortality, cardiovascular, and renal events early after pregnancy. Recurrent, severe, and early‐onset preeclampsia further increases this risk.

**Nonstandard Abbreviations and Acronyms**
CONCEPTION: Cohort of Cardiovascular Diseases in Pregnancy
HDP: hypertensive disorders of pregnancy
SNDS: French National Health Insurance Information System database (Système National des Données de Santé)

**Clinical Perspective**

**What Is New?**

- Hypertensive disorders of pregnancy drastically increase the risk of mortality, cardiovascular, and renal events early after pregnancy.
- This risk is further increased when preeclampsia is recurrent, severe, early‐onset, or combined with hypertension or with small‐for‐gestational‐age infants.

**What Are the Clinical Implications?**

- Health care professionals should inform women with a history of hypertensive disorder of pregnancy of their increased cardiovascular risk.
- These women should visit their physician at least once a year for checks on blood pressure and metabolic factors and to encourage them adopt a healthier lifestyle.

Hypertensive disorders of pregnancy (HDP) are a group of diseases that include chronic hypertension predating pregnancy, gestational hypertension, and preeclampsia.^1^ These conditions, which affect approximately 1 pregnant woman in 10, are a leading cause of mother and child morbidity, and are considered major public health issues worldwide.^2^, ^3^ Although they typically resolve after delivery, many studies have reported that women with a history of HDP are more likely to develop chronic hypertension,^4^, ^5^, ^6^ cardiovascular disease,^7^, ^8^, ^9^, ^10^ kidney disease,^11^ heart failure,^9^, ^10^ and to die of cardiovascular causes^12^ later in life. In 2011, the growing body of evidence on these risks prompted the American Heart Association to recognize that having a history of HDP constitutes a cardiovascular risk factor.^13^

Many of the findings from the above‐mentioned studies were based on large population‐based databases, which limit the risk of selection bias and provide sufficient statistical power to identify associations between HDP and rare events.^14^, ^15^, ^16^, ^17^, ^18^, ^19^, ^20^, ^21^ Some of the studies also investigated the association between these outcomes and HDP recurrence,^9^, ^16^ the severity and onset time of preeclampsia,^4^, ^12^, ^21^, ^22^, ^23^ preeclampsia combined with small‐for‐gestational‐age (SGA) infants,^18^, ^21^ and preeclampsia combined with chronic hypertension.^21^, ^24^

However, the substantial differences in the study populations, investigation periods, and methods used (study design, type of HDP and outcomes studied, definition and identification of HDP and outcomes, follow‐up time, adjustment for confounders) in these studies translate into a high level of heterogeneity in meta‐analyses compiling their results.^8^, ^9^, ^10^, ^11^, ^12^ Moreover, the associations between HDP and cardiovascular events are mainly studied over the long term; few studies have looked at the first decade after pregnancy.

To the best of our knowledge, no study to date has made a comprehensive analysis of the association between HDP (type, severity, precocity, recurrence) and either mortality or cardiovascular, cerebrovascular, and renal events.

In this context, the present study aimed to evaluate the impact of HDP occurrence, as well as the impact of recurrent, early onset, and severe gestational hypertension and preeclampsia on both mortality and on a wide range of cardiovascular and renal outcomes in France, using data from the CONCEPTION (Cohort of Cardiovascular Diseases in Pregnancy) nationwide cohort study.

## METHODS

### Data Source

CONCEPTION is a prospective cohort study designed to investigate hypertensive disorder and cardiovascular event epidemiology in women who gave birth in France after 22 weeks of gestation between January 1, 2010 and December 31, 2018.^25^ It is based on data from the French National Health Insurance Information System database (Système National des Données de Santé [SNDS]), and includes >4.5 million women and 7.1 million childbirths. The study's methodology and characteristics are described elsewhere.^3^, ^4^ The SNDS database contains comprehensive information on all health care expenditures reimbursed by the national health insurance system for the entire French population (≈66 000 000 people). It comprises 2 information sources: the National Hospital Discharge Database, which records information on public and private hospital stays, including diagnoses, using *International Classification of Diseases, Tenth Revision* (*ICD‐10*) codes, and the Interscheme Consumption Data Mart, which records information on out‐of‐hospital care (eg, medicines, imaging, outpatient medical care).

In line with the French national regulations and ethics committee, participant consent and institutional review board approval were not required for this study. Santé Publique France, the French public health agency, has full and permanent access to the Système National des Données de Santé (governmental deliberation number 2016‐316, October 13, 2016). We cannot share national health data system data, because they are only available on a secure portal. Authorization to access this portal needs registration and clearance.

### Study Population

For the present study, we included all women in CONCEPTION who gave birth for the first time in France after 22 weeks of gestation between January 1, 2010 and December 31, 2018. Before 22 weeks of gestation, miscarriages are not systematically documented in medical records in France, hence their exclusion from the present analyses. For women who gave birth by vaginal delivery, the parity was directly available in patients' medical records. Women who had a cesarean section were considered primiparous if no previous childbirth was identified since 2006. We excluded women aged <15 and >49 years, those with a medical history of cardiovascular disease since 2006, and those with an invalid date of pregnancy. Study follow‐up spanned from the beginning of the first pregnancy to December 31, 2021 (ie, 3‐year minimum follow‐up for those who delivered for the first time at the end of 2018).

### Hypertensive Disorders of Pregnancy

For all women included in the final population, we identified all HDP during the first and subsequent pregnancies until December 31, 2021, with algorithms combining diagnoses coded during hospital stays (*ICD‐10*) and reimbursements for specific drugs.

Chronic hypertension predating the first pregnancy was defined as receiving prescribed antihypertensive drugs on at least 3 different dates (2 different dates if at least 1 large package of 90 pills was dispensed) between 1 year preceding the first pregnancy and 20 weeks of gestation. The definition of new‐onset chronic hypertension was the same throughout the study follow‐up (including interpregnancy periods), but with the dispensing of antihypertensive drugs over a rolling period of 1 year. For each pregnancy, women were also defined as having preexisting chronic hypertension if they were hospitalized with a primary diagnosis of preexisting chronic hypertension (*ICD‐10* codes: O10, O11) during pregnancy or postpartum. We considered the date of the first dispensing of antihypertensive drugs as the date of diagnosis of chronic hypertension. Women diagnosed with this condition before or during the study were considered to have it for the rest of the follow‐up period irrespective of where or not antihypertensive drugs were interrupted.

Gestational hypertension was defined as either receiving at least 1 antihypertensive drug between 20 weeks of gestation and 6 weeks postpartum, or hospitalization with a diagnosis of gestational hypertension (*ICD‐10* code O13) in the absence of previous chronic hypertension. To avoid potential misclassification, women reimbursed for antihypertensive drugs and hospitalized with preterm labor as a primary diagnosis (*ICD‐10* codes O47, O60.0–O60.2) were excluded from the gestational hypertension group, because antihypertensive treatment may have been prescribed for this situation.

Preeclampsia, eclampsia, and hemolysis, elevated liver enzymes, and low platelets syndrome were defined as hospitalization, with *ICD‐10* diagnosis codes O14, O15, and 014.2, respectively. We considered preeclampsia to be severe when recorded as such (code 014.1), or when it was complicated by eclampsia or hemolysis, elevated liver enzymes, and low platelets syndrome. For all other situations, it was considered mild. Furthermore, preeclampsia was defined as early onset when it occurred before 34 weeks of gestation, and late onset otherwise. Finally, for singleton pregnancies with a delivery in or after 2013, preeclampsia combined with SGA infant was defined as a birth weight inferior to the 10th percentile for gestational age and sex.^26^ This year was chosen because data on the fetus' weight and sex pre‐2013 were not exhaustive.

### Outcomes

The study outcomes were mortality and the occurrence of the following cardiovascular, cerebrovascular, and renal events during follow‐up: stroke (all types, ischemic, hemorrhagic), acute coronary syndrome, peripheral artery disease, heart failure, rhythm or conduction disorder, pulmonary embolism, and chronic kidney disease. These events (ie, except mortality) were identified by the primary diagnoses coded during hospitalization (Table S1). The date of hospitalization was considered to be the date of the event. With regard to mortality, the date of death was directly available in the SNDS. Causes of death coded with the *ICD‐10* classification were available only until 2017. Accordingly, for the 2010 to 2017 period, we identified cardiovascular deaths if the primary diagnosis of death belonged to Chapter IX of the *ICD‐10* classification (I00–I99: diseases of the circulatory system).

### Study Population Characteristics and Covariates

At baseline, preexisting diabetes was identified using an algorithm based on the dispensing of 3 antidiabetic drugs on 3 different dates in the year preceding the first pregnancy, or on 2 dates if at least 1 large package (ie, 90 pills) of antidiabetic drugs was dispensed. Obesity and tobacco smoking were identified by *ICD‐10* codes during hospitalization, and by the dispensing of prescribed nicotine replacement therapy before or during pregnancy for tobacco smoking. People who benefitted from Universal Medical Coverage, a social benefit in France for those whose income is below a certain ceiling, at baseline were defined as living in social deprivation.

### Statistical Analysis

We first described the characteristics (number and percentages for categorical variables, mean and standard deviation for continuous variables) of women at baseline, and the rates of HDP and cardiovascular events during follow‐up. We then calculated the rates of each event during follow‐up for all women combined, and then separately for women with gestational hypertension and those with preeclampsia. The associations between HDP and the different outcomes were assessed by hazard ratios (HRs) calculated using crude and adjusted Cox proportional regression models. In all models, occurrences of HDP were treated as time‐varying variables. The follow‐up started at the beginning of the first pregnancy and was censored at December 30, 2021 or death. In a first set of models, we considered the following HDP exposures (with relevant modalities): total gestational hypertension, total preeclampsia, gestational hypertension recurrence (no gestational hypertension, gestational hypertension only once, gestational hypertension at least twice), preeclampsia recurrence (no preeclampsia, preeclampsia only once, preeclampsia at least twice). In a second set of models, we focused on the following preeclampsia features: preeclampsia severity (no preeclampsia, mild preeclampsia, severe preeclampsia), early‐onset preeclampsia (no preeclampsia, late preeclampsia, early preeclampsia), preeclampsia associated with SGA infant (no preeclampsia, preeclampsia with no SGA infant, preeclampsia with SGA infant; this analysis was restricted singleton pregnancies with a delivery in or after 2013), and preeclampsia combined with chronic hypertension (no preeclampsia, preeclampsia with no chronic hypertension, preeclampsia with chronic hypertension). The modality preeclampsia with hypertension included both women with chronic hypertension predating pregnancy and women who developed chronic hypertension after preeclampsia occurred.

Associations of each HDP type with each outcome were modeled separately; women could have >1 incident outcome and be exposed to >1 HDP. If a woman had preeclampsia, we did not take into account gestational hypertension in subsequent pregnancies, for we considered that the risk associated with preeclampsia exposure outweighs that associated with gestational hypertension. Maternal age in years was used as the time scale instead of time on study in all models, because this method is better at reducing the risk of bias than subsequently adjusting or stratifying for maternal age.^27^ The models were adjusted for social deprivation, obesity, tobacco smoking, and history of diabetes. HRs with their 95% CIs are presented in forest plots. The proportional hazard assumption was tested by assessing the correlation between Schoenfeld residuals and time. All statistical analyses were conducted using SAS software. Figures were created using the forest plot package in R.

## RESULTS

**Figure 1:**
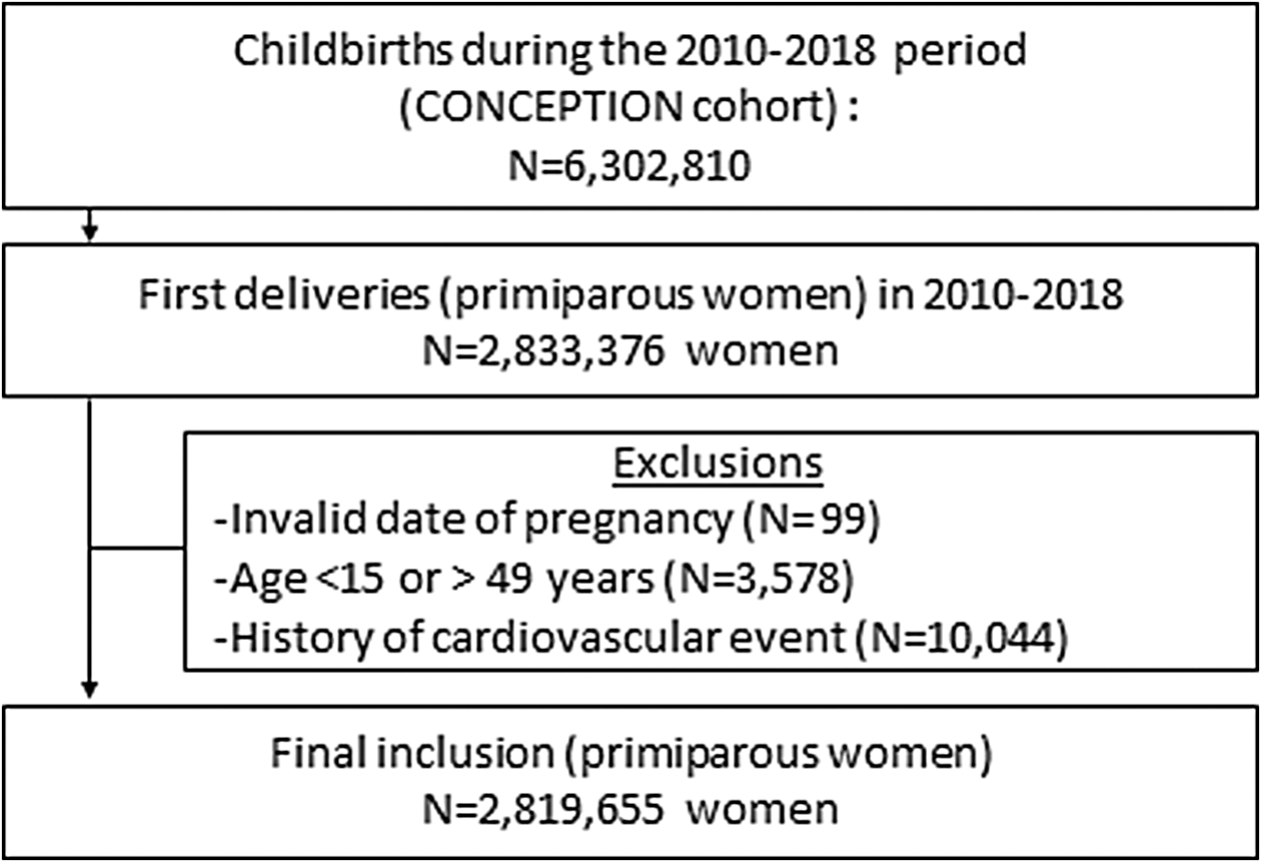
Study flowchart. CONCEPTION indicates Cohort of Cardiovascular Diseases in Pregnancy.

Among the 6 302 810 deliveries in France in 2010 to 2018 and included in the CONCEPTION cohort, we identified 2 833 376 first‐time births (Figure 1). We excluded 99 women with discrepancies between the dates of subsequent pregnancies, 3578 women at extreme ages, and 10 044 women with a history of cardiovascular, neurovascular, or renal disease after 2006. The final population included 2 819 655 women, accounting for 4 913 570 childbirths between 2010 and 2021.

Table 1 presents the characteristics of the study population. At baseline (ie, start of the first pregnancy), the mean age was 28.3 years, 13.7% of women lived in social deprivation, 4.1% had obesity, 0.5% had diabetes, and 8.9% were smokers. The mean follow‐up duration was 8.42 years (median, 8.53 years). During follow‐up, 6.2% had gestational hypertension at least once, and 3.6% had preeclampsia at least once.

**Table 1:** Population Characteristics at Baseline and During Follow‐Up (N=2 819 655)

	N or mean	% or SD
At baseline		
Age, y	28.30	5.36
Social deprivation	386 661	13.7%
Chronic hypertension	41 308	1.5%
Obesity	116 450	4.1%
Diabetes	14 250	0.5%
Smoking	250 490	8.9%
During follow‐up		
Follow‐up duration	8.42	2.60
No. of pregnancies	1.74	0.73
Only 1	1 130 537	40.1%
2	1 344 994	47.7%
≥3	344 124	12.2%
Hypertensive disorders of pregnancy (at least 1)		
Gestational hypertension	173 460	6.2%
Preeclampsia	100 348	3.6%
Mild and late preeclampsia	62 006	2.2%
Severe or early preeclampsia	46 855	1.7%
Cardiovascular event during follow‐up		
Stroke	4598	0.16%
Ischemic stroke	3131	0.11%
Hemorrhagic stroke	1642	0.06%
Acute coronary syndrome	1640	0.06%
Heart failure	1358	0.05
Pulmonary embolism	5168	0.18%
Peripheral arterial disease	544	0.02%
Kidney disease	1917	0.07%
Cardiac rhythm and conduction disorder	7219	0.26%
All‐cause deaths	3658	0.13%
Cardiovascular deaths (until 2017)	193	0.01%

Crude numbers and rates of cardiovascular events across the follow‐up according to HDP type are presented in Table 2. During follow‐up, 21 708 women (0.77%) experienced at least 1 cardiovascular event, and 3658 died (all causes). Apart from rhythm and conduction disorder, all outcomes were more likely in women who had gestational hypertension at least once. This likelihood was even higher in women who had preeclampsia at least once. Specifically, 0.14% and 0.51% of those who had gestational hypertension and preeclampsia, respectively, developed chronic kidney disease during follow‐up. Similarly, 0.12% and 0.32%, respectively, developed heart failure. Moreover, the mean time‐to‐event was shorter (1 to 2 years) in women who had preeclampsia (Table S2).

**Table 2:** Crude Rates and Numbers of Cardiovascular Events During Follow‐Up According to the HDP

HDP	Total		Gestational hypertension		Preeclampsia	
	N	%	N	%	N	%
Stroke	4598	0.16%	415	0.24%	396	0.39%
Ischemic stroke	3131	0.11%	281	0.16%	237	0.24%
Hemorrhagic stroke	1642	0.06%	153	0.09%	180	0.18%
Acute coronary syndrome	1640	0.06%	196	0.11%	186	0.19%
Peripheral arterial disease	544	0.02%	49	0.03%	73	0.07%
Heart failure	1358	0.05%	214	0.12%	318	0.32%
Pulmonary embolism	5168	0.18%	362	0.21%	318	0.32%
Chronic kidney disease	1917	0.07%	236	0.14%	511	0.51%
Rhythm/conduction disorder	7219	0.26%	783	0.45%	307	0.31%
All‐cause death	3658	0.13%	287	0.17%	223	0.22%
Cardiovascular death (until 2017)	193	0.01%	23	0.01%	27	0.03%

Women could contribute to >1 incident event and be exposed to both gestational hypertension and preeclampsia. Consequently, the numbers of events in each row and column may intersect, and no statistical test was performed. HDP indicates hypertensive disorders of pregnancy.

**Figure 2:**
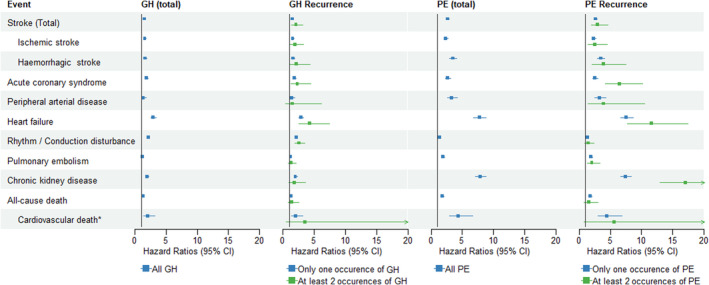
Adjusted hazard ratios of cardiovascular events according to gestational hypertension or PE and its recurrence. All models were adjusted for diabetes history, social deprivation, obesity, and tobacco use. Maternal age was used as the time scale. Exposures were treated as time‐varying variables. *The follow‐up time was censored at December 31, 2017 in models analyzing cardiovascular death, because causes of death were available only for the 2010 to 2017 period. GH indicates gestational hypertension; and PE, preeclampsia.

After adjustment for confounders, and treating HDP as time‐varying variables, the HRs (95% CIs) for cardiovascular disease in women who had gestational hypertension at least once during follow‐up were 1.57 (1.42–1.74) for stroke (all types), 1.58 (1.39–1.78) for ischemic stroke, 1.62 (1.37–1.91) for hemorrhagic stroke, 1.85 (1.59–2.15) for acute coronary syndrome, 1.37 (1.02–1.83) for peripheral artery disease, 2.92 (2.53–3.38) for heart failure, 2.19 (2.04–2.36) for rhythm and conduction disorder, and finally 1.25 (1.12–1.39) for pulmonary embolism (Figure 2; Table S3). This same subgroup was also 1.98 (1.73–2.27) times more likely to develop chronic kidney disease, 1.33 (1.18–1.5) times more likely to die (all causes), and 2.09 (1.35–3.24) times more likely to die of cardiovascular death. HRs, except for chronic kidney disease, were higher in women who had gestational hypertension at least twice than in those who had it only once, but the difference was not statistically significant.

In women who had preeclampsia at least once, the HRs (95% CIs) were 2.67 (2.4–2.96) for stroke (all types), 2.33 (2.04–2.66) for ischemic stroke, 3.48 (2.97–4.07) for hemorrhagic stroke, 2.7 (2.31–3.16) for acute coronary syndrome, 3.3 (2.56–4.26) for peripheral artery disease, 7.75 (6.8–8.82) for heart failure, 1.35 (1.2–1.51) for rhythm and conduction disorder, and 1.92 (1.71–2.15) for pulmonary embolism.

This subgroup was 7.91 (7.11–8.80) times more likely to develop chronic kidney disease during follow‐up, 1.78 (1.55–2.04) times more likely to die (all causes), and 4.37 (2.88–6.63) times more likely to die of cardiovascular death. HRs, except for all‐cause death, were higher in women who had preeclampsia at least twice than in those who only had it once. The difference was only statistically significant for acute coronary syndrome and chronic kidney disease.

**Figure 3:**
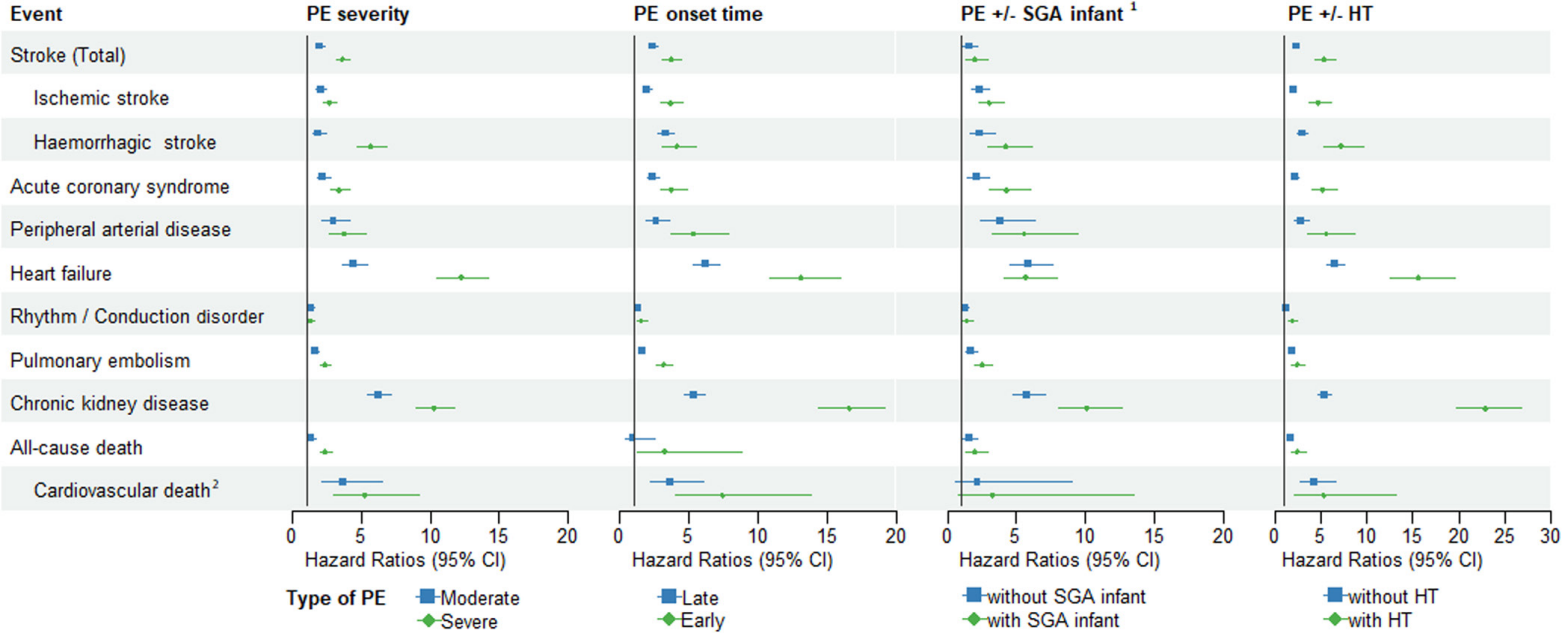
Adjusted hazard ratios of cardiovascular events according to the severity and earliness of PE and its association with SGA infant and chronic hypertension. All models were adjusted for diabetes history, social deprivation, obesity, and tobacco use. Maternal age was used as the time scale. Exposures were treated as time‐varying variables. Models explain PE with/without SGA infant. Birth weight was available only for singleton births between 2013 and 2018. The follow‐up time was censored at December 31, 2017 in models explaining cardiovascular death, because causes of death were available only for the 2010 to 2017 period. HT indicates hypertension; PE, preeclampsia; and SGA, small for gestational age.

Figure 3 shows the HRs for each outcome according to the severity and the onset time of preeclampsia, and according to preeclampsia combined with SGA infant and with hypertension. Women who had severe preeclampsia were significantly more likely to develop stroke (all types and hemorrhagic), heart failure, pulmonary embolism, and chronic kidney disease, and more likely to die (all cause) than women who had mild preeclampsia. Women who had early‐onset preeclampsia were significantly more likely to develop stroke (all types and ischemic), acute coronary syndrome, peripheral artery disease, heart failure, pulmonary embolism, and chronic kidney disease than women who had late‐onset preeclampsia. Women whose preeclampsia was combined with SGA infant were significantly more likely to develop chronic kidney disease than women whose preeclampsia was not combined with SGA infant. Finally, women whose preeclampsia was combined with chronic hypertension were significantly more likely to have a stroke (all types), acute coronary syndrome, heart failure, and chronic kidney disease than women whose preeclampsia was not combined with chronic hypertension.

## DISCUSSION

In this large population‐based study of >2.8 million women and 4.9 million childbirths, gestational hypertension and preeclampsia were strongly associated with occurrence of cardiovascular, cerebrovascular, and renal events, and with all‐cause and cardiovascular mortality early in the women's lives. Women who had preeclampsia at least twice were even more likely to have acute coronary syndrome or chronic kidney disease. Severe preeclampsia, early onset preeclampsia, preeclampsia in combination with SGA infant, and preeclampsia in combination with chronic hypertension all strengthened these associations, especially for chronic kidney disease.

Our findings are consistent with those reported in previous population‐based and cohort studies. In their systematic review and meta‐analysis, Wu et al found that women with a history of preeclampsia had a 2‐fold higher risk of coronary heart disease, stroke, and cardiovascular disease death, and a 4‐fold higher risk of heart failure.^10^ Lo et al found that women with a history of gestational hypertension were 1.8 times more likely to have cardiovascular diseases, coronary heart disease, and heart failure (but not stroke).^9^ Barrett et al found that the risk of chronic kidney disease was 2.1 times higher in women who had preeclampsia, and 1.5 times higher in women who had gestational hypertension.^11^

Many studies have focused on the excess risk induced by severe and early‐onset preeclampsia,^12^, ^16^, ^22^, ^28^ preeclampsia combined with SGA infant,^18^, ^28^ preeclampsia combined hypertension,^24^ and by HDP recurrence^16^, ^29^, ^30^, ^31^ on a wide variety of outcomes. Nonetheless, these studies are heterogeneous in terms of their populations, designs, HDP studied, and outcomes, making it difficult to compare and compile their results. This is reflected in Pittara et al's systematic review, where 73% of the 90 associations collected for long‐term outcomes after preeclampsia had a high or very high degree of heterogeneity.^8^ Our study provides a comprehensive overview of the different impacts of all types of HDP, their recurrence, onset time, severity, combination with SGA infant, and combination with hypertension, on the occurrence of 11 cardiovascular, cerebrovascular, and renal events, including all‐cause and cardiovascular death. Many of these associations have never been previously assessed.

The question as to whether HDP have a causal relationship with cardiovascular disease onset or only reveal an underlying higher cardiovascular risk is a controversial one. Many authors consider pregnancy as a stress test for life, unmasking physiological susceptibility to cardiovascular disease,^32^, ^33^ whereas others highlight that cardiac and hemodynamic impairment caused by preeclampsia can persist long after the end of pregnancy.^34^, ^35^ Interestingly, we found that having gestational hypertension at least twice was not associated with a higher risk of cardiovascular events than once‐off gestational hypertension occurrence, whereas women who had preeclampsia at least twice had a significantly higher risk of acute coronary syndrome and chronic kidney disease than women who had it only once. Similarly, severe and early‐onset preeclampsia was associated with an even higher risk of cardiovascular events. These results support the hypothesis of a direct and persistent effect of preeclampsia on the cardiovascular system. Recently, another study based on data from the CONCEPTION cohort reported that preeclampsia duration increased the risk of developing hypertension in the first years following childbirth.^4^ This association was only partly explained by the difference of gestational age. Moreover, echocardiographic studies found that hemodynamic alterations are correlated with severe and early‐onset preeclampsia.^36^ Together, these findings suggest that, in women with a history of preeclampsia, the longer and more severe the exposure to antiangiogenic factors, the higher is the long‐term cardiovascular risk.

In view of the above evidence, the postpartum period should be regarded as a window of opportunity to implement interventions aimed at assessing and reducing cardiovascular risk in women who had an HDP. Most national and international guidelines recommend that these women be provided counseling on this excess risk and on the importance of the medical follow‐up throughout their lives.^37^, ^38^, ^39^ Specifically, they should visit a physician at least once a year to check their blood pressure and metabolic factors. Recent studies showed that home self‐monitoring by mothers could be a promising alternative to improve blood pressure control.^40^ The International Federation of Gynecology and Obstetrics recently promoted tailored lifestyle modifications in women with a history of HDP, such as a heart‐healthy diet (consumption of fruits and vegetables, Dietary Approaches to Stop Hypertension diet), physical activity, and body mass index control.^41^ Breastfeeding is also effective in reducing future cardiovascular risk.^42^ Unfortunately, the implementation of these educational measures is still largely insufficient. Qualitative studies and surveys have reported that many women are not given information about their increased cardiovascular risk after an HDP.^43^, ^44^ Even more worrying is the lack of awareness of physicians themselves about the association between HDP and cardiovascular events reported in other studies.^45^, ^46^ Moreover, information on a history of HDP is not always transmitted to primary care providers (general practitioners, obstetricians/gynecologists) who offer postpartum follow‐up.^47^ This communication gap translates into a lost opportunity for women and should be tackled.

The present study has several strengths. First, thanks to its prospective population‐based design, made possible by the use of France's national medico‐administrative database (SNDS), we were able to comprehensively include all hospital deliveries in France over a period of 10 years. Accordingly, the risk of inclusion bias was limited, because out‐of‐hospital deliveries represent only 0.6% of all deliveries in France.^48^ Second, thanks to the large number of women included, we had excellent statistical power to be able to estimate, in a holistic manner, the independent associations between HDP types, their recurrence, onset time, and severity on a wide range of cardiovascular outcomes, including estimating the association between rare exposure (eg, preeclampsia recurrence) and rare events (eg, peripheral artery disease). To the best of our knowledge, these associations have never previously been assessed in a single population using only 1 method. Moreover, many of the associations we explored have never been reported in previous studies. Finally, the use of a method with time‐varying exposures provided us with a comprehensive overview of the occurrence of these events from the beginning of the first pregnancy to the end of follow‐up.

The study also has limitations. First, the SNDS database was initially designed for medico‐administrative purposes and therefore lacks clinical data; we identified chronic hypertension and gestational hypertension through the dispensing of antihypertensive drugs and diagnosis codes. Accordingly, we probably missed some cases of untreated gestational hypertension and hypertension not coded during hospital stays, most probably mild forms of these diseases, which might have diluted the risk of the events. Similarly, we were not able to identify cardiovascular events that did not lead to hospitalization. This is particularly true for diseases commonly treated in ambulatory care, such as rhythm disorder or kidney disease. Having said that, insufficient identification of events was likely to be of the same order in HDP‐exposed and nonexposed women; consequently, the lack of these data should not skew the associations between HDP and the various events considered. For the same reason, covariates such as obesity and tobacco use were insufficiently identified, and we had no data on ethnicity, alcohol consumption, or weight gain/loss during follow‐up. These missing data may have led to residual confounding. Finally, we did not have information on other causes of missing follow‐up such as emigration. Nonetheless, we believe that these causes are rare, evenly distributed in the exposed and nonexposed groups, and unlikely to cause a significant bias in the association between HDP and outcomes.

## CONCLUSIONS

This population‐based prospective study showed that all HDP were associated with the occurrence of cardiovascular, cerebrovascular, and renal events and with all‐cause and cardiovascular mortality in the early years after the first pregnancy. Severe, early‐onset, and recurrent preeclampsia were associated with an even higher cardiovascular risk in these women. In light of these results, and with the aim of improving women's health and life expectancy, it would appear crucial for policymakers to improve awareness about the increased risk of cardiovascular and renal events in women with a history of HDP, to encourage them to visit their physician at least once a year for checks on blood pressure and metabolic factors, and to urge them to follow healthier lifestyles.

## Sources of Funding

This study was funded by the Société Française d'Hypertension Artérielle, Fondation de Recherche sur l'Hypertension Artérielle, and the Fédération Française de Cardiologie through the call for scientific projects Thematic Grant 2019: Cardiovascular Diseases in Women. The funders had no role in the study design, data collection, data analysis, decision to publish, or drafting of the article.

## Disclosures

S.K. reports, outside the submitted work, nonfinancial support from Lilly France, Novonordisk, Novartis Pharma, Roche Diabetes Care, Lifescan, Abbott France, Sanofi, ViiV Healthcare, Servier, and Becton Dickinson, and personal fees from Icomed, Pascaleo, BT3SI, and M3global Research. J.B. has received, outside the submitted work, compensation as speaker/chairman/consultant/educational activities from Astra‐Zeneca, Bayer, ElKendi, Hikma, Leurquin, Omron, Organon, Sanofi Aventis, Vivactis, and Vivoptim. The remaining authors have no disclosures to report.

## Ethical Approval

In accordance with French national regulations and the ethics committee, participant consent was not required for this study. Santé Publique France, the French public health agency, has full and permanent access to the SNDS (governmental deliberation number 2016‐316, October 13, 2016).

## Supporting information

Tables S1–S3
