# Supplementary material for: Early Mortality, Cardiovascular, and Renal Diseases in Women's Lives Following Hypertensive Disorders of Pregnancy: The Prospective Nationwide Study CONCEPTION
Source: J Am Heart Assoc. 2024 Apr 2;13(8):e033252. doi: 10.1161/JAHA.123.033252 (PMC11262502; doi:10.1161/JAHA.123.033252)
Supplement: Supplementary file 1 — Tables S1–S3 [file JAH3-13-e033252-s001.pdf]

SUPPLEMENTAL MATERIAL

Table S1. ICD-10 codes used for the identification of cardiovascular and renal events.

Cardiovascular and renal events	ICD-10 codes	
	Primary diagnosis	Accompanying diagnosis
Stroke	I60, I61, I62, I63, I64, O225, O873	
Ischemic stroke	I63, I64, O225, O873	
Haemorrhagic stroke	I60, I61, I62	
Acute coronary syndrome	I200, I21, I22, I23	
Peripheral arterial disease	I702, I739, I740, I743, I744, I745	
Heart failure	I50, I11, I130, I132	
Heart failure (other identification)*	I139, J81, K761	I50, I11, I130, I132
Rhythm or conduction disorder	I44, I45, I47, I48, I49	
Pulmonary embolism	I26, O882	
Chronic kidney disease	N18, I12, I13, E102, E112, I122, I132,E142, Z490, Z940, N118, N1119	

*Heart failure was identified by a code of heart failure either as primary diagnosis, or as accompanying diagnosis when associated with a primary diagnosis of pulmonary edema, liver congestion or hypertensive heart disease,

Table S2. Mean time-to-event by hypertensive disorders of pregnancy

By Gestational Hypertension

	Mean time-to-event (years)			Mean difference
	Total	No GH	GH	
Stroke	5.0	5.0	4.8	0.2
Ischemic stroke	5.0	5.1	4.6	0.4
Hemorragic stroke	4.8	4.7	4.9	-0.2
Acute coronary syndrome	6.0	6.1	5.1	0.9
Peripheral arterial disease	5.6	5.6	5.8	-0.2
Heart failure	3.7	3.8	3.2	0.7
Rythm/ conduction disturbance	4.9	5.0	3.8	1.2
Pulmonary embolism	4.4	4.5	4.0	0.5
Chronic kidney disease	4.4	4.5	3.6	0.9
All-cause death	4.7	4.7	4.3	0.4

By Pre-eclamspia

	Mean time-to-event (years)			Mean difference
	Total	No pre-eclampsia	Pre-eclampsia	
Stroke	5.0	5.2	3.6	1.6
Ischemic stroke	5.1	5.2	4.0	1.1
Hemorragic stroke	4.7	4.9	2.9	2.1
Acute coronary syndrome	6.0	6.1	4.7	1.4
Peripheral arterial disease	5.6	5.8	4.6	1.2
Heart failure	3.7	4.2	2.0	2.2
Rythm/ conduction disturbance	5.0	5.0	4.2	0.8
Pulmonary embolism	4.4	4.5	3.1	1.5
Chronic kidney disease	4.2	4.6	3.1	1.5
All-cause death	4.7	4.7	3.7	1.1

GH=Gestational Hypertension

Table S3. Adjusted Hazard Ratios of cardiovascular events according to HDP type and recurrence.

	Adjusted Hazard Ratios (95% Confidence Interval)										
	Stroke (all type)	Ischemic stroke	Haemoragic stroke	Coronary syndrome	Peripheral arterial disease	Heart Failure	Rythm/Conduction disturbance	Pulmonary embolism	Chronic kidney disease	death	Cardiovascular death[2]
**HDP recurrence**											
**Gestational Hypertention recurrence**											
No GH	Ref	-	-	-	-	-	-	-	-	-	-
Only one GH	1.55 (1.4-1.72)	1.56 (1.38-1.77)	1.59 (1.34-1.89)	1.83 (1.57-2.14)	1.36 (1.01-1.84)	2.87 (2.47-3.33)	2.18 (2.02-2.34)	1.25 (1.12-1.39)	1.99 (1.73-2.29)	1.33 (1.17-1.5)	2.05 (1.31-3.21)
At least 2 GH	2.09 (1.38-3.18)	1.97 (1.16-3.32)	2.15 (1.07-4.31)	2.3 (1.2-4.44)	1.53 (0.38-6.14)	4.3 (2.49-7.43)	2.56 (1.87-3.51)	1.3 (0.78-2.15)	1.8 (0.9-3.6)	1.43 (0.81-2.51)	3.52 (0.5-25.07)
**Pre-eclampsia recurrence**											
No PE	Ref	-	-	-	-	-	-	-	-	-	-
Only one PE	2.65 (2.38-2.95)	2.65 (2.38-2.95)	3.46 (2.95-4.06)	2.53 (2.15-2.98)	3.28 (2.53-4.25)	7.55 (6.6-8.62)	1.34 (1.19-1.51)	1.91 (1.7-2.15)	7.44 (6.66-8.31)	1.79 (1.55-2.05)	4.33 (2.83-6.61)
At least 2 PE	2.95 (1.9-4.59)	2.95 (1.9-4.59)	3.88 (2.02-7.48)	6.5 (4.17-10.12)	3.9 (1.45-10.46)	11.64 (7.74-17.49	1.44 (0.87-2.4)	2.04 (1.25-3.34)	17.03 (12.97-22.38)	1.56 (0.81-3.01)	5.67 (0.79-40.66
**Pre-eclampsia subtypes**											
**Severity**											
No PE	Ref	-	-	-	-	-	-	-	-	-	-
Mild PE	1.98 (1.7-2.31)	2.07 (1.73-2.48)	1.88 (1.44-2.46)	2.21 (1.78-2.75)	2.98 (2.13-4.17)	4.46 (3.64-5.46)	1.37 (1.18-1.58)	1.61 (1.37-1.89)	6.24 (5.41-7.19)	1.35 (1.11-1.65)	3.7 (2.09-6.56)
Severe PE	3.61 (3.15-4.13)	2.69 (2.23-3.24)	5.66 (4.7-6.82)	3.36 (2.73-4.13)	3.75 (2.65-5.32)	12.23 (10.51-14.2	1.32 (1.1-1.57)	2.36 (2.02-2.76)	10.27 (8.99-11.74)	2.36 (1.97-2.82)	5.25 (3.02-9.15)
**Precocity**											
No PE	Ref	-	-	-	-	-	-	-	-	-	-
Late PE	2.38 (2.11-2.69)	1.97 (1.68-2.32)	3.31 (2.77-3.95)	2.37 (1.97-2.86)	2.63 (1.92-3.61)	6.21 (5.32-7.26)	1.29 (1.13-1.47)	1.59 (1.38-1.83)	5.38 (4.7-6.16)	0.91 (0.34-2.47)	3.42 (2.03-5.74)
Early PE	3.72 (3.1-4.47)	3.66 (2.93-4.58)	4.12 (3.07-5.54)	3.74 (2.9-4.83)	5.35 (3.66-7.82)	13.15 (10.85-15.9	1.57 (1.25-1.98)	3.16 (2.62-3.82)	16.64 (14.44-19.17)	3.26 (1.2-8.85)	7.5 (4.01-14)
**Small for gestational age (SGA)[1]**											
No PE	Ref	-	-	-	-	-	-	-	-	-	-
PE without SGA	1.58 (1.13-2.21)	2.33 (1.76-3.07)	2.36 (1.6-3.48)	2.09 (1.43-3.07)	3.87 (2.35-6.35)	5.89 (4.53-7.66)	1.24 (0.98-1.58)	1.68 (1.32-2.14)	5.79 (4.71-7.11)	1.58 (1.13-2.21)	2.18 (0.53-9.03)
PE with SGA	1.98 (1.36-2.88)	3.04 (2.23-4.14)	4.23 (2.93-6.11)	4.29 (3.04-6.05)	5.57 (3.27-9.47)	5.68 (4.06-7.94)	1.43 (1.07-1.9)	2.54 (1.97-3.27)	10.12 (8.06-12.7)	1.98 (1.36-2.88)	3.28 (0.79-13.56
**Hypertension (HT)**											
No PE	Ref	-	-	-	-	-	-	-	-	-	-
PE without HT	2.29 (2.03-2.58)	1.99 (1.71-2.32)	2.96 (2.48-3.54)	2.18 (1.81-2.63)	2.82 (2.1-3.79)	6.55 (5.66-7.58)	1.27 (1.13-1.44)	1.85 (1.64-2.1)	5.38 (4.71-6.14)	1.67 (1.44-1.95)	4.21 (2.68-6.61)
PE with HT	5.34 (4.37-6.52)	4.71 (3.64-6.09)	7.18 (5.34-9.65)	5.22 (4.03-6.76)	5.58 (3.6-8.66)	15.61 (12.47-19.5	1.9 (1.44-2.5)	2.42 (1.83-3.22)	22.93 (19.67-26.74)	2.44 (1.79-3.31)	5.32 (2.13-13.25

GH= Gestational Hypertension ; HDP= Hypertensive Disorders of Pregnancy ; HT= Hypertension ; SGA= Small for Gestational Age ; PE= Pre-eclampsia

[1]For singleton births born between 2013 and 2018

[2]The follow-up time was censored the 31/12/17 for these two outcomes, since causes of death were available only for the 2010-2017 period.
